# A spatiotemporal reconstruction of the 1630 plague epidemic in Milan

**DOI:** 10.1016/j.isci.2023.106704

**Published:** 2023-04-20

**Authors:** Massimo Galli, Riccardo Nodari, Matteo Perini, Ester Luconi, Luca Fois, Folco Vaglienti, Claudio Bandi, Elia Biganzoli, Francesco Comandatore

**Affiliations:** 1 Department of Biomedical and Clinical Sciences, University of Milan, Milan, Italy; 2 Romeo ed Enrica Invernizzi Paediatric Research Centre, Department of Biomedical and Clinical Sciences, University of Milan, Milan, Italy; 3 Department of Environmental Health Sciences, Mailman School of Public Health, Columbia University, New York, NY, USA; 4 Department of Humanities, Section of Historical and Geographical Science, University of Pavia, Pavia, Italy; 5 Department of Historical Studies, University of Milan, Milan, Italy; 6 Romeo ed Enrica Invernizzi Paediatric Research Centre, Department of Biosciences, University of Milan, Milan, Italy

**Keywords:** Health sciences, Biological sciences, Microbiology

## Abstract

In 1630, a devastating plague epidemic struck Milan, one of the most important Italian cities of that time, deeply affecting its demography and economy for decades. The lack of digitized historical data strongly limits our comprehension of that important event. In this work, we digitized and analyzed the Milan death registers of 1630. The study revealed that the epidemic evolved differently among the areas of the city. Indeed, we were able to group the parishes of the city (comparable with modern neighborhoods) in two groups based on their epidemiological curves. These different epidemiological progressions could reflect socio-economical and/or demographic features specific of the neighborhoods, opening questions about the relationship between these features and the evolution of epidemics in the pre-modern period. The study of historical records, like the one presented here, can help us to better understand European history and pre-modern epidemics.

## Highlights

• Digital reconstruction of one of the most important epidemic in Italian history
• City neighborhoods showed different epidemic progressions
• Historical data can increase our understanding of pre-modern epidemics

## Graphical abstract

**Figure:**
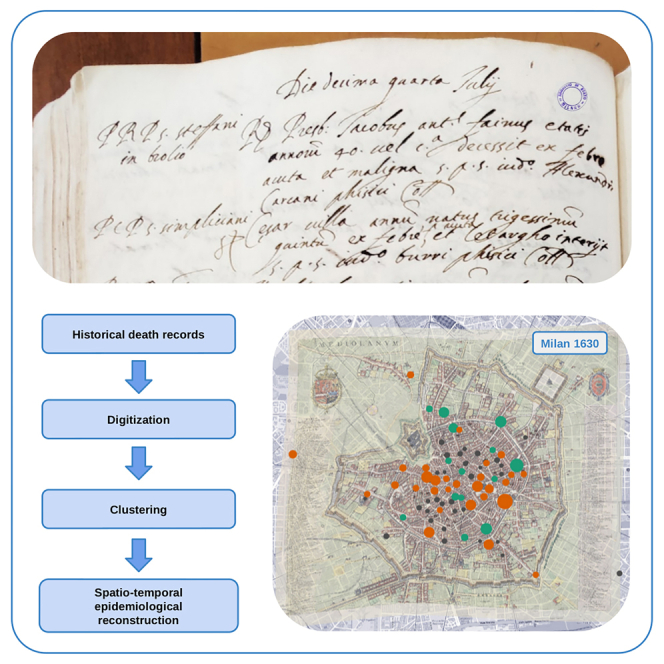

## Introduction

In 1630, Milan suffered the last of the 11 major plague epidemics that affected the city since 1361, that was particularly devastating and left a profound mark on the demography, economy, and culture of the city and northern Italy, contributing to the country’s economic decline in the XVII century.^1^ The epidemic was brought to Italy in 1629 during a war that struck both urban and rural communities, and while famine ravaged the country.^2^^,^^3^ It has been estimated that there were about two million victims in northern Italy, with a total mortality rate of 30%–35%,^2^ and an estimated median rate of as high as 40% in the 26 largest cities of that region.^1^

After the first cases were officially recorded in the city in October 1629, the epidemic seemed to disappear during the winter, re-emerging in March 1630.^4^^,^^5^ At the end of March 1630, Milan’s Lazzaretto held fewer than 300 people,^6^ but by the end of June that number had become thousands.^7^^,^^9^^,^^8^ Two of the most important chroniclers of the time, Giuseppe Ripamonti and Alessandro Tadino, affirmed that the number of plague deaths increased after the San Carlo Borromeo procession on 11 June 1630: “*… eadem ab motis D. Caroli Reliquijs, & solicitato veluti Coelo terribilior multò violentiorque facta fuerit ….*” (Once the relics of San Carlo were moved and Heaven was solicited, the facts became much more terrible and violent), *Ripamonti page 66;*^4^ “*Finite queste solennità molto più si accese il fuoco della peste in tutte le parti della Città*” (At the end of these solemnities, the fire of the plague flared even more throughout the city.), *Tadino page 108*.^5^

Unfortunately, only a few epidemiology studies have been published so far on the 1630 plague epidemic in northern Italy,^10^^,^^11^^,^^12^ and no one regarding the epidemic in the city of Milan. An important, albeit underused, detailed source of real historical epidemiological data is the *Mortuorum Libri* (Book of the Dead) of Milan, a large series of registers daily compiled between 1450 and 1801 by physician of that time which contains the clinical and geographical information about all the people that died in the city.^13^^,^^14^

In this paper, we performed a spatiotemporal reconstruction of the plague epidemic that devastated Milan in 1630 using the data contained in the Milan death registers.

## Results

### Digitization of death registers (Mortuorum Libri)

The digitized death registers were analyzed to obtain the relevant epidemiological information from the records (see data availability statement). Of the 8,152 recorded deaths found, 5,261 (64.5%) were reported as caused by plague. Excluding three isolated cases between January and early March 1630, all the plague deaths occurred between 24 March and 30 December (71.2% of the total number of deaths occurring in the same period). The number of plague deaths in each parish varied widely, with the parish of *San Stefano in Brolo* having the highest number (592).

### Daily incidences of plague and not-plague deaths

**Figure 1:**
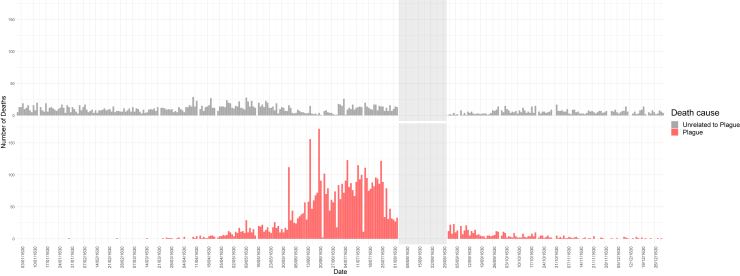
Barplots of the number of deaths per day in the city of Milan in 1630 The daily number of total plague deaths is shown in the red histogram on the bottom, while the daily number of those not related to plague is in the gray histogram at the top. The shaded area (4–30 August) indicates the period for which the death register is unavailable. More details are shown in Video S1 (Dynamic Spatiotemporal representation of the plague epidemic).

Figure 1 shows the daily incidence of recorded deaths. The absence of records between 4 and 30 August is due to the lack of the register reporting the deaths occurred in that period. The frequency of plague deaths per parish over time and the relative absolute number of plague and plague-unrelated deaths are shown in Video S1 (Dynamic spatiotemporal representation of the plague epidemic).

Video S1. Dynamic Spatiotemporal representation of the plague epidemic On the left, the points placed on the 1629 Milan historical map represent the geolocalized parishes; the size of the point changes over time as the relative number of daily deaths and the color of each point depends on the percentage of plague deaths: from black (0% plague deaths) to red (100% plague deaths); the path of the San Carlo procession appears on 11 June 1630 (and the video stops for a few seconds). On the right, the curve of the total plague deaths is reported in red and that of the plague-unrelated deaths in gray; two light-gray vertical lines slide the graphs day by day.

**Figure 2:**
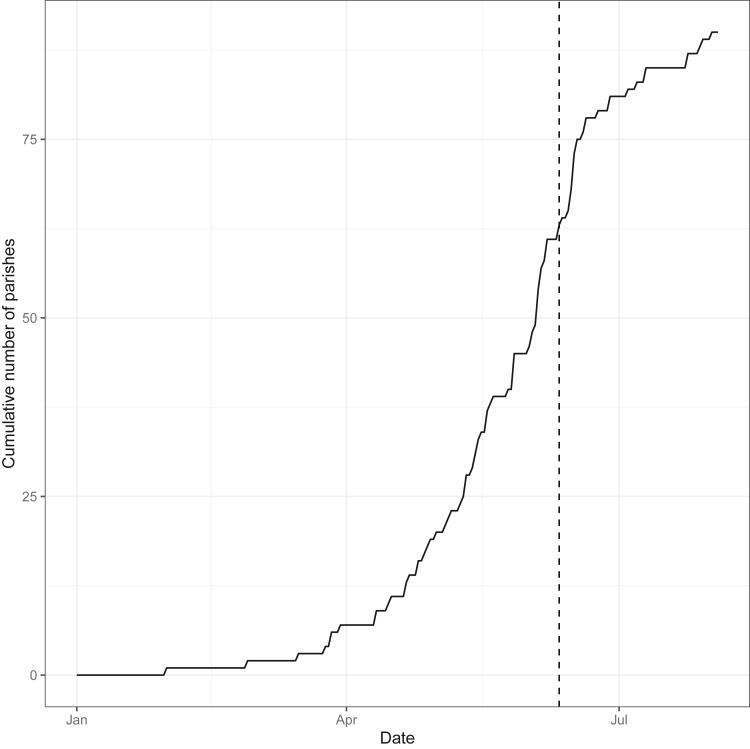
Cumulative number of parishes with one plague death over time For each day between 1 January 1630 and 4 August 1630, the number of parishes with at least one plague death is plotted. The vertical dashed line refers to 11 June 1630, the date of the San Carlo procession.

As shown in Figure 2, for 63 out of 94 parishes (67%), the first plague death occurred before 11 June, the day of the San Carlo procession.

### Parish clustering

**Figure 3:**
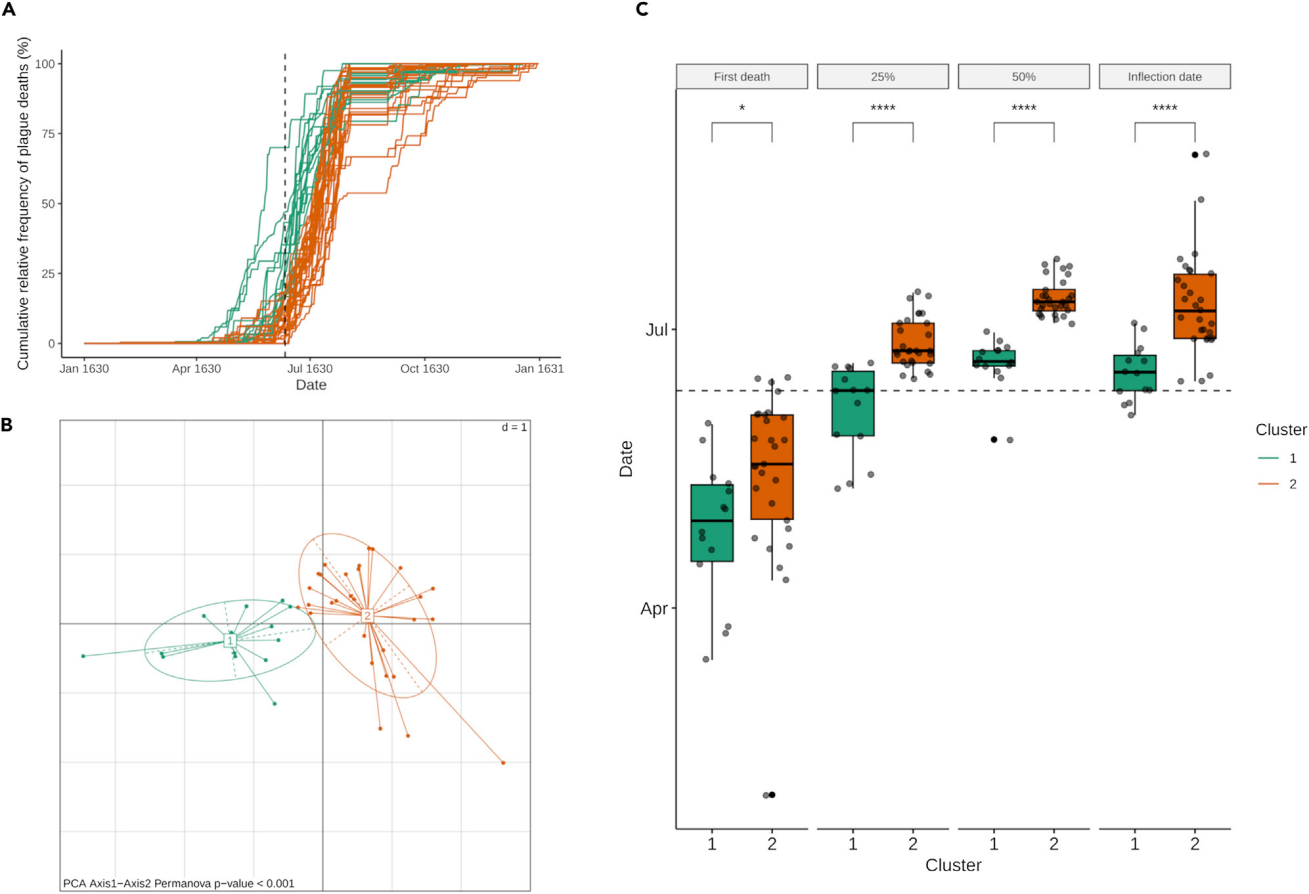
Clustering of the parishes on plague cumulative relative frequency curves Analysis of the cumulative relative frequency curves of the 43 parishes with, on average, at least one plague death every two weeks of epidemic (i.e., 21 total plague deaths). The curves were subjected to fuzzy clustering analysis and principal component analysis (PCA) which identify two clusters (labeled “Cluster 1” and “Cluster 2”). In all the plots, cluster 1 is colored in green and cluster 2 in orange. (A) Plague cumulative relative frequency curves of the 43 parishes colored on the basis of the cluster: on the x axes the date and on the y axes the cumulative number of plague deaths. The dotted vertical gray line indicates the day of the San Carlo procession (11 June 1630). (B) PCA using the Euclidean distance on the plague cumulative relative frequency curves. (C) From left to right, boxplots of the dates for which the parishes of the two clusters reached first plague death, 25% of total plague deaths, 50% of total plague deaths and the inflection point of the curves (i.e., the date at which the cumulative curve changes concavity, corresponding to the epidemic peak). In each boxplot, the dates for the two clusters were compared using a Mann-Whitney U test (‘∗’ p value <0.05; ‘∗∗∗∗’ p value <0.0001). The boundaries of the whiskers of the boxplot are based on the 1.5 Interquartile range (IQR) value. The dotted line indicates the date of the San Carlo procession.

The 43 parishes with more than 21 recorded deaths were grouped based on their cumulative epidemiological curves (Figure 3A). The optimal number of clusters determined by silhouette analyses was two. K-means clustering divided the parishes into two groups that respectively included 14 (cluster 1) and 29 parishes (cluster 2). Permanova testing confirmed the significance of the obtained clusters (p value <0.001) (Figure 3B).

Figure 3C shows the comparisons of parameters relative to the epidemiological curves of the parishes of the two clusters. The parishes of cluster 1 experienced the first plague death significantly earlier than those of cluster 2 (Mann-Whitney U test; median cluster 1: 1630-04-29, median cluster 2: 1630-05-18; p value <0.05). Similarly, the parishes of cluster 1 reached 25% (median cluster 1: 1630-06-11, median cluster 2: 1630-06-24) and 50% (median cluster 1: 1630-06-20, median cluster 2: 1630-07-10) of their total plague deaths earlier than the parishes of cluster 2 (Mann-Whitney U test; p value <0.0001). Lastly, the parishes of the two clusters significantly differ also for the date of the inflection point (the date at which the curve changes concavity) of their epidemiological curves (Mann-Whitney U test; median cluster 1: 1630-06-17, median cluster 2: 1630-07-07; p value <0.0001). Moreover, the two clusters showed no significant difference in both global deaths (Mann-Whitney U test; cluster 1: 2,640, cluster 2: 4,362; p value = 0.39) and total plague deaths (Mann-Whitney U test; cluster 1: 1,625, cluster 2: 2,979; p value = 0.99).

### Parishes geolocalization

Seventy-nine of the 94 parishes recorded in the registers were successfully geolocalized and accounted for 93% of all the reported plague deaths; the remaining 15 parishes accounted for 0.99% of all the reported plague deaths. The parish information was not present in the registers for 6% of all plague deaths.

**Figure 4:**
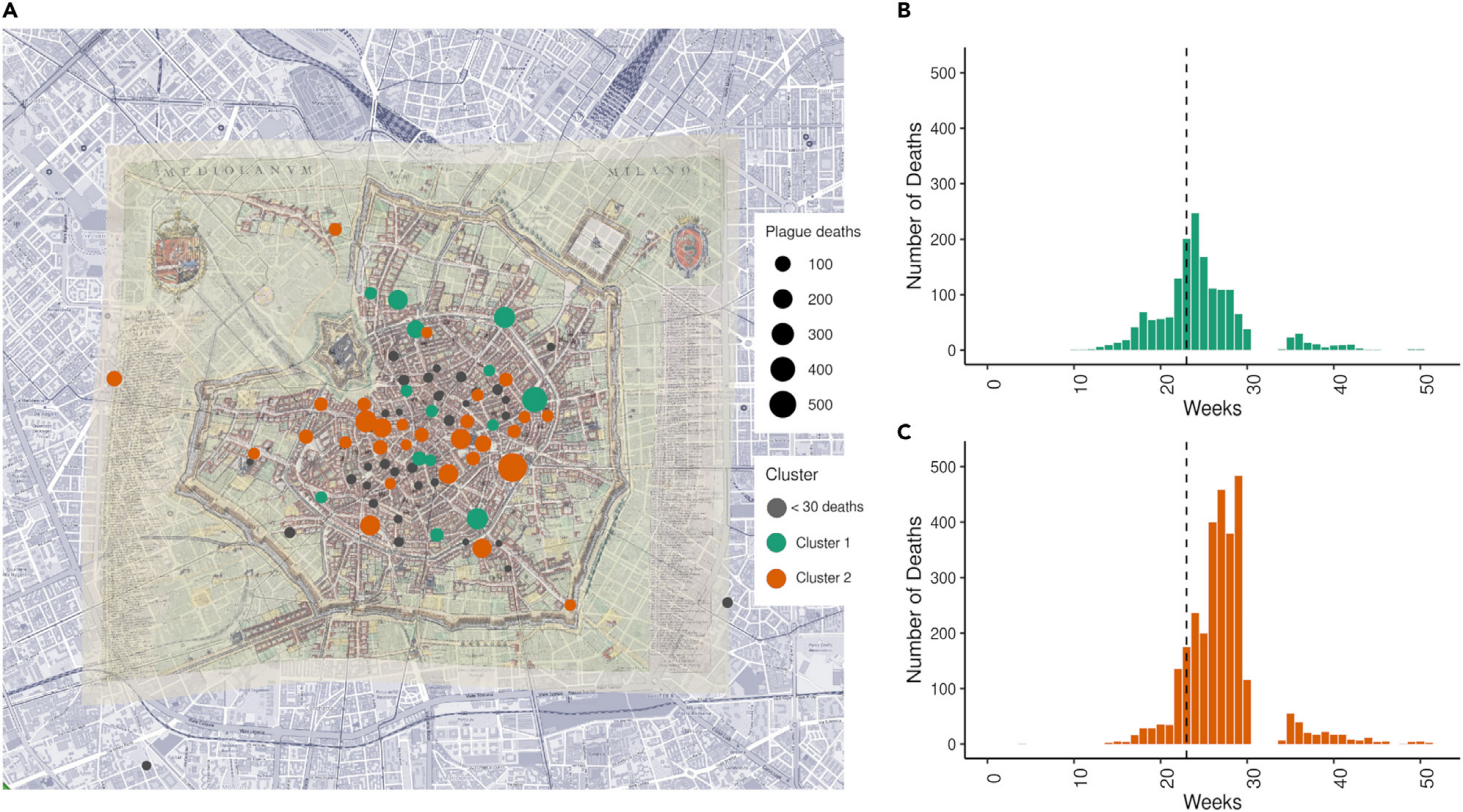
Clusters spatiotemporal distribution (A) Parishes localization on an historical 1629 Milan map. In green, parishes of cluster 1; in orange, those of cluster 2; in gray, the parishes with less than 21 total plague deaths (not used in the clustering analysis). (B) Total weekly plague deaths of cluster 1 parishes; the dashed line shows the date of the procession (11 June 1630). (C) Total weekly plague deaths of cluster 2 parishes; the dashed line shows the date of the procession (11 June 1630).

The position of the 79 georeferenced parishes is shown on the historical map of the city of Milan (Figure 4A). The weekly incidences of plague deaths for the parishes of cluster 1 and cluster 2 are reported in Figures 4B and 4C, respectively. Lastly, the median distance from the city center of the parishes of cluster 1 is not significantly different from those of cluster 2 (one-tailed Mann-Whitney U test; median cluster 1: 790 m, cluster 2: 515 m; p value = 0.12).

## Discussion

Toward the end of 1629, northern Italy was the scene of the War of Succession of Mantua, an episode in the Thirty Years' War. The army that crossed the Alps to besiege the city of Mantua brought with them plague.^15^ The disease rapidly spread in northern Italy, also reaching the city of Milan, where it remained apparently silent until the spring of the following year, when the epidemic began.^5^ The chroniclers of the time reported that the number of plague deaths drastically increased in summer, in particular after the San Carlo procession (11 June 1630), a religious event that involved most of the city inhabitants. This plague epidemic is considered one of the most relevant in Italian history, as narrated in the masterpiece Italian novel “The Betrothed”^16^ by Alessandro Manzoni. In this study, we reconstructed this important epidemic by applying modern analytic tools to the original records collected during 1630 in the Milan death registers.

These registers contain individual records of more than 8,000 Milan citizens who died in the city during 1630, of which >5,000 died of plague. For each deceased citizen, the registers also contain information about their localization in the city, reported as the parishes relative to their house (comparable with the modern city neighborhoods). We found that ∼80% of the plague deaths occurred between the beginning of June and the end of July. The analysis of plague deaths cumulative curves revealed that the parishes can be grouped in two clusters. The parishes of cluster 1 were affected by the plague epidemic before those of cluster 2. Cluster 1 parishes registered a lower median date for the first plague deaths and reached 25% and 50% of the total plague deaths before those of cluster 2. Furthermore, cluster 1 parishes reached their plague epidemic peak (i.e., the date of the inflection value of the epidemic cumulative curve) before cluster 2 parishes. We can exclude that this effect is due to a different total number of plague deaths; indeed, the parishes of the two clusters do not significantly differ for global deaths and total plague deaths.

Coherently with what was reported by the chroniclers of the time, June 1630 was an important moment for the evolution of the epidemic. Before 11 June, the day of the San Carlo procession, about 70% of all the parishes recorded at least one plague death, suggesting that the epidemic had reached most of the city. Nevertheless, only 876 out of 5,261 recorded plague deaths (17%) occurred before this event. The same period was also important for the epidemic progression in the parishes of the two clusters: while cluster 1 parishes reached their peak in that period, cluster 2 parishes experienced a drastic increase in the number of deaths in the weeks immediately after.

Our analysis clearly showed the existence of two groups of parishes with different epidemic progressions, possible due to socio-economical and/or demographic differences (e.g., different hygienic conditions). Unfortunately, at the state of the art, no detailed information about the population structure and characteristics of the different parishes of Milan in that period is available to test this hypothesis.^17^

Due to the extreme rarity of detailed historical/epidemiological records on past events in literature, several pieces of information about this important plague epidemic remain unknown. This work is the first detailed epidemiological reconstruction of the Milan plague epidemic of 1630, obtained using modern analytical tools on historical data. Anyway, this work just scratched the surface of the information contained in the death registers of Milan, which cover the period from the first half of the 15th century to the beginning of the 19th century. Studying historical records opens a window into our past, allowing us to understand important information about pre-modern human societies and their pathogens.

### Limitations of the study

The main limitation of the work presented here is that in the registers used for the analysis are reported only the deaths occurred at home or in city streets. Unfortunately, to our knowledge, the data concerning the deaths that occurred in the lazaretto (the hospital reserved for people suspected to be infected), hospitals, and convents were not preserved or were unavailable. Nevertheless, the data relating to deaths in the city and streets are probably the most suitable for investigating the spatiotemporal dynamics of the epidemic.

## STAR★Methods

### Key resources table

**Table:** 

REAGENT or RESOURCE	SOURCE	IDENTIFIER
**Deposited data**		

Raw and analyzed data	This paper	https://github.com/RiccardoND/Milan-plague-epidemic-paper-1630
Original code	This paper	https://github.com/RiccardoND/Milan-plague-epidemic-paper-1630

**Software and algorithms**		

R	R: A language and environment for statistical computing.	https://www.R-project.org/; RRID:SCR_001905
QGIS	QGIS Geographic Information System	http://www.qgis.org; RRID:SCR_018507

### Resource availability

#### Lead contact

Further information and requests for resources and code should be directed to and will be fulfilled by the lead contact, Riccardo Nodari (riccardo.nodari@unimi.it).

#### Materials availability

This study did not generate new unique reagents.

### Experimental model and subject details

#### Digitization of death registers (Mortuorum Libri)

The data used for the analyses were retrieved from the *Mortuorum Libri*, a *corpus* of more than three hundred registers recording the deaths occurring in the city between 1450 and 1801 that is kept in the State Archive of Milan.^14^^,^^18^ Each death report includes demographic information (name, surname, age, gender, titles, or particular status of the deceased person), the parish (i.e., territorial entity comparable with modern city neighborhoods) of the city in which the person lived, the main cause of death as certified by the city’s appointed medical officers (and manually curated by an expert physician). For the purposes of this study, the three available registers for 1630 were consulted and digitized; one register could not be found in the public archive and so the records concerning deaths between 4 August 1630 and 31 August 1630 are missing. An expert paleographer translated and transcribed the Latin texts, and all the data were organized in a tabular file.

### Method details

#### Daily incidence of plague and not-plague deaths

All the recorded deaths were used to reconstruct the epidemiological histogram of the number of daily deaths caused by plague and other causes as certified by the city’s appointed medical officers (masking the period between 4 August 1630 and 31 August 1630).

#### Daily number of parishes with at least one plague death

To study the diffusion of the plague in the city, the first plague death for each parish was considered for the period between 1 January 1630 and 4 August 1630 (indeed, the absence of the information after this date interruption of the dataset made the rest of the records not reliable for this analysis). For each day, the number of parishes with at least one plague death was computed. This information allowed to figure out the spreading of the disease in the city.

#### Parish clustering

The parishes were clustered using the cumulative curve of plague deaths. In order to obtain reliable cumulative curves, only the parishes with more than 21 total plague deaths were considered (i.e., more than one plague death every two weeks of epidemic). For each selected parish, the plague death cumulative relative frequency curve (i.e., the curve reporting the number of plague deaths counted until each day, from here “plague cumulative curves”) was computed. The plague cumulative curves were subjected to Principal Component Analysis (PCA) using the Euclidean distance and parishes were grouped using the unsupervised clustering algorithm K-means^19^ (after selecting the number of clusters with silhouette analysis). The significance of the clusters was also tested using the Permanova test.^20^

Furthermore, the Mann-Whitney test was applied to compare the dates at which the parishes of the clusters reached the 25% of total plague deaths, 50% of total plague deaths, the dates of the first plague death, and the dates corresponding to the inflection point of the plague cumulative curves (the date at which the cumulative curve changes concavity, corresponding to the epidemic peak). Then, both the number of global deaths (due to any causes) and total plague deaths for the two clusters were compared using a Mann-Whitney U test.

For each cluster, the histogram of the weekly plague deaths was produced.

#### Parishes geolocalisation

Each parish (i.e., territorial entity comparable with modern city neighborhoods) was geographically localized based on the position of its relative church. The longitude and latitude (in decimal degrees, North-East) of each parish was determined using Quantum GIS software^21^ and the position of the related church was established based on a digitized historical map of Milan dated 1629^22^ or by using historical text.^23^ To visualize the geographical distribution of the clusters, all parishes were plotted on an historical map of Milan^22^ and colored depending on the corresponding cluster. Then, the distance of each parish from the city center (defined as the centroid of the area delimited by the city’s medieval walls) were computed using Quantum GIS software.^21^ The median distance of the parishes of the clusters were then compared using the Mann-Whitney U test, excluding the parishes located outside the city walls (i.e., "S. Trinità", "S. Rocco", "S. Pietro in Sala").

### Quantification and statistical analysis

All the analyses were performed using R version 4.1.2.^24^ Details of statistical analysis are provided within the relevant method details section. For all results, significance was defined as follows: ∗p < 0.05, ∗∗p < 0.01, ∗∗∗p < 0.001, ∗∗∗∗p < 0.0001.

## Data Availability

• data have been deposited at https://github.com/RiccardoND/Milan-plague-epidemic-paper-1630 and are publicly available as of the date of publication. A link to code has been included in the key resources table. • All original code has been deposited at https://github.com/RiccardoND/Milan-plague-epidemic-paper-1630 and is publicly available as of the date of publication. A link to code has been included in the key resources table. • Any additional information required to reanalyse the data reported in this paper is available from the lead contact upon request.
